# Hemorrhage from the Tongue, Successfully Treated with Tincture of Matico

**Published:** 1851-07

**Authors:** 


### ARTICLE XVII.

### *Hemorrhage from the Tongue, successfully treated with Tincture of Matico.*

Mr. Welch reports the following case as having occurred in the London Hospital during his dressership; a fruiterer, aged thirty-four years, states that on the 6th instant he received a blow upon the chin, whilst the tongue was protruding from the mouth, which caused that organ to be wounded by the teeth. Hemorrhage followed, so copious and uncontrollable, as to render it necessary to apply for surgical assistance. After many unsuccessful attempts to suppress the bleeding, for three days, by several surgeons, he was brought to this hospital in a very weak state, and placed under the care of Mr. Luke. The wound was at first rubbed over with a piece of the nitrate of silver, but the bleeding still continued. A saturated solution of alum was then applied by means of pieces of lint, but still without success ; and at last the tincture of matico was had recourse to, and used in the same way as the solution of alum. Fortunately it had the desired effect of arresting the hemorrhage permanently.

The patient states, that whenever bleeding from his nose had taken place it had generally continued for several days ; also that when he was once bled from the arm it was found very difficult to stop the blood, which oozed through the compress and bandage for nearly four days. He further states that he was a patient in this hospital some years ago, on account of hemorrhage from the urethra, which continued for the space of seven days, and was at last stopped by keeping a large catheter for a long time in the urethra. He was also, it appears, at another time in this hospital, in consequence of having received a wound on his hand, which bled for several days, notwithstanding continual pressure was kept up by compresses, thus showing a very strong hemorrhagic diathesis. On inquiry, it is found that all the members of his family have the same hemorrhagic disposition, particularly his father.

The patient, after having been one week in the hospital, without any return of the hemorrhage, was discharged, with directions to take tonic medicines, and to use such other means as would be likely to have a tendency to strengthen his constitution and improve his general health.

